# A Potential Relationship Between HALP Score and In‐Hospital Mortality in Acute Heart Failure

**DOI:** 10.1002/clc.70108

**Published:** 2025-03-01

**Authors:** Akbulut Muge, Izci Cenan, Ozyuncu Nil, K. Esenboga

**Affiliations:** ^1^ Department of Cardiology Ankara University School of Medicine Altındağ Turkey

**Keywords:** heart failure, HALP score, in‐hospital mortality

## Abstract

**Introduction:**

Acute heart failure (AHF) is associated with a dismal prognosis that is even poorer than the majority of cancer types. Therefore, clinical indicators that can aid in determining the prognosis of heart failure are of interest. Multiple risk prediction tools with varying sensitivity and specificities have been introduced before. In the current study, we aimed to evaluate whether the HALP score could accurately predict in‐hospital mortality in patients with AHF.

**Methods:**

We evaluated the medical records of a total of 153 patients admitted to our institution between August 2016–May 2018 for acute heart failure. The patients were divided into two groups: Group 1 (patients who died during hospital admission) and Group 2 (patients who were discharged from the hospital). The HALP score was calculated as: hemoglobin (g/L) x albumin (g/L) x lymphocytes (/L)/platelets (/L) for each patient. The two groups were compared in terms of HALP scores. The receiver operator characteristic (ROC) curve was utilized to assess the predictive performance of HALP on in‐hospital mortality in AHF.

**Results:**

Patients who died during admission had lower HALP scores compared with the patients who were discharged uneventfully. A ROC curve analysis was performed to predict the optimal cut‐off value of the HALP score. The area under the curve (AUC), sensitivity, specificity, and the cut‐off value were 0.650, 43%, 57%, 21,5 respectively (*p* = 0.014).

**Conclusion:**

Despite all evolving treatment modalities, heart failure‐related mortality rates remain high. Prompt recognition of patients with an unfavorable prognosis is vital for the timely implementation of disease‐modifying therapeutic interventions. The HALP score, being a readily calculable tool, serves as an effective means to pinpoint individuals at a heightened risk of in‐hospital mortality. We believe that the HALP score holds promise as a practical tool for predicting in‐mortality among patients admitted for AHF.

## Graphical abstract

HALP Score Predicts In‐hospital Mortality in Patients With Acute Heart Failure

- Acute heart failure (AHF) has a dismal prognosis. Multiple risk prediction tools with varying sensitivity and specificities have been introduced before. We aimed to evaluate whether the HALP score could accurately predict in‐hospital mortality in patients with AHF.
- 153 patients admitted for acute heart failure:
- Group 1: patients who died during hospital admission
- Group 2: patients who were discharged from the hospital
- HALP score was calculated as: hemoglobin (g/L) x albumin (g/L) × lymphocytes
- (L)/platelets (/IL).
- HALP score was lower in patients who have died during admission. HALP = 21.5 (AUC = 0.650, *p* = 0.014) predicts mortality in patients admitted for AHF with a sensitivity of 40% and specificity of 60%.
- The HALP score, being a readily calculable tool, serves as an effective means to pinpoint individuals at a heightened risk of in‐hospital mortality.

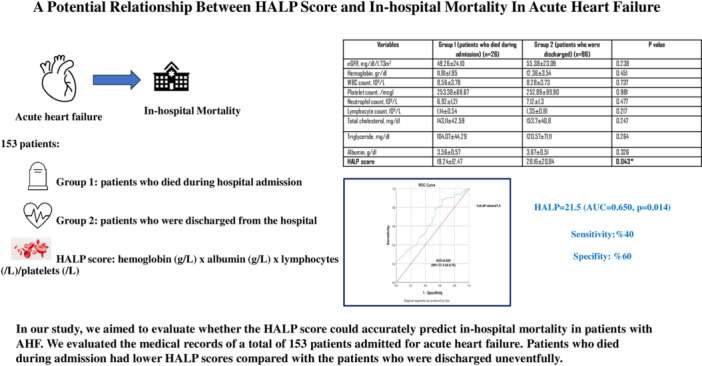

## 1 Introduction

Acute heart failure (AHF) is characterized by new or worsening signs and/or symptoms that are predominantly related to pulmonary or systemic congestion, necessitating urgent intervention [1]. Globally, AHF remains the leading cause of hospitalizations among adults. In the general population, AHF prevalence ranges between 0.3% and 2%, whereas it approaches 25% in patients older than 75 years old [2, 3]. In Turkey, the estimated prevalence of heart failure is 6.9% within the general population [4].

AHF is associated with a dismal prognosis, often surpassing that of many cancers. Despite rigorous efforts in management, rates of hospital readmission and residual congestion upon discharge remain high. In American and European registries, in‐hospital mortality rates reach 4%–7%, with post‐discharge mortality rates reported as high as 36% [5, 6, 7]. Consequently, identifying clinical indicators that can aid in determining the prognosis is of significant interest, and numerous risk prediction tools with varying sensitivities and specificities have been introduced [8, 9, 10, 11, 12, 13].

Recently, a novel index based on hemoglobin, albumin, lymphocyte, and platelet levels has been reported to have good accuracy in predicting outcomes across various cancer types [14, 15]. Kocaoglu et al. [16] further investigated the predictive validity of the HALP score in determining mortality in patients with AHF. They concluded that the traditional HALP score did not correlate with mortality in AHF patients. However, they developed a novel score, the modified HALP score (mHALP), which was significantly associated with 3‐month mortality in AHF.

In the current study, we aimed to evaluate the utility of the HALP score in predicting in‐hospital mortality among patients with AHF.

## 2 Methods

### 2.1 Study Population

In this retrospective cohort study, we evaluated the medical records of a total of 153 patients admitted to our institution between August 2016–May 2018 for acute heart failure. The acute heart failure definition was based on the current European Society of Cardiology clinical practice guidelines for heart failure in 2021 [17]. We excluded patients with active infections, end‐stage malignancy, and hepatic failure from the study. The analysis included a final count of 122 patients. The patients were divided into two groups: Group 1 (patients who died during hospital admission) and Group 2 (patients who were discharged from the hospital).

### 2.2 Data Collection

The demographic and clinical data of the patients were obtained from the medical records. The HALP score was calculated as: hemoglobin (g/L) x albumin (g/L) x lymphocytes (/L)/platelets (/L). In‐hospital mortality was defined as all‐cause mortality before discharge. The study protocol was approved by the local ethics (Ankara University School of Medicine) committee (Date: 24/09/2021, Decision No: 2021000335‐1) and was performed in concordance with ethical rules and principles of the Declaration of Helsinki. The ethics committee waived the requirement for participant consent due to the retrospective nature of the study.

### 2.3 Statistical Analyses

The analyses were conducted with the Statistical Package for Social Sciences for Windows 10.0 (SPSS Inc., Chicago, IL, United States). Continuous variables were shown as mean ± standard deviation (SD), whereas categorical ones were shown as percentages (%). The categorical variables were compared with the c^2^ test, whereas the continuous variables were analyzed using the Independent Samples *t*‐test. ROC curve analysis was used to measure the power of the HALP score. Univariate and multivariate analyses were performed to identify the predictors of OS; variables with *p* < 0.2 in the univariate analysis were included in the multivariate analysis. Statistical significance was accepted for a two‐sided value of *p* < 0.05.

## 3 Results

A total of 122 patients were included in the final analysis. The in‐hospital mortality rate of the patient cohort was 21.3%. The baseline characteristics of the two groups are presented in Table 1. Patients who died during admission (group 1) had lower HALP scores, compared to patients who were discharged uneventfully. The two groups were similar in terms of other baseline characteristics.

**Table 1:** The baseline demographic and clinical characteristics of the patients.

Variables	Group 1 (patients who died during admission) (*n* = 26)	Group 2 (patients who were discharged) (*n* = 96)	*p* value
***Age (years)***	74.96 ± 7.66	72.47 ± 9.59	0.226
***Men n***	19	57	0.201
***Smoker n***	4	10	0.481
***Co‐morbidities (n)***			
HT	23	81	0.602
DM	19	54	0.121
AF	4	24	0.301
***Medications (n)***			
ACEI	16	57	0.155
Statins	23	77	0.332
BB	20	73	0.925
P2Y12 inhibitors	10	35	0.851
Oral anticoagulants	5	12	0.379
ASA	21	58	0.054
***History of PCI (n)***	15	45	0.513
***History of CABG (n)***	12	28	0.102
***History of TIA/CVA (n)***	2	3	0.297
***Etiology of heart failure (n)***			
Ischemic	15	45	0.513
Dilated	11	51	
***NYHA class (n)***			0.780
II	1	2	
III	8	35	
IV	17	59	
***Laboratory values***			
eGFR, mg/dl/1.73m^2^	49.26 ± 24.10	55.38 ± 23.09	0.238
Hemoglobin, gr/dl	11.81 ± 1.95	12.36 ± 3.54	0.451
WBC count, 10^9^/L	8.56 ± 3.78	8.28 ± 3.73	0.737
Platelet count,/mcgl	253.38 ± 69.67	252.89 ± 99.90	0.981
Neutrophil count, 10^9^/L	6.92 ± 1.21	7.12 ± 1.3	0.477
Lymphocyte count, 10^9^/L	1.14 ± 0.54	1.35 ± 0.81	0.217
Total cholesterol, mg/dl	143.11 ± 42.59	153.7 ± 40.8	0.247
HDL cholesterol, mg/dl	36.84 ± 10.80	36.19 ± 9.95	0.773
LDL cholesterol, mg/dl	85.11 ± 34.24	94.11 ± 33.20	0.226
Triglyceride, mg/dl	104.07 ± 44.29	120.57 ± 71.11	0.264
Albumin, g/dl	3.56 ± 0.57	3.67 ± 0.51	0.326
NT‐proBNP, pcg/mL	3329.75 ± 6192.79	2853.09 ± 3589.67	0.620
C‐reactive protein, mg/dl	26.27 ± 25.51	29.62 ± 38.60	0.682
HALP score	19.24 ± 12.47	28.16 ± 20.84	0.043*
***Echocardiographic values***			
LA AP diameter (cm)	4.91 ± 0.52	4.97 ± 0.57	0.661
LVEF, (%)	27.82 ± 7.65	28.08 ± 8.89	0.911
sPAP, mmHg	57.11 ± 16.92	54 ± 12.83	0.310
Mitral E velocity (m/sec)	0.87 ± 0.32	0.74 ± 0.23	0.243

Abbreviations: ACEI, angiotensin converting enzyme inhibitor; AF, atrial fibrillation; ASA, acetylsalicylic acid; BB, beta‐blocker; CABG, coronary artery bypass graft surgery; CVA, cerebrovascular accident; DM, diabete mellitus; eGFR, estimated glomerular filtration rate; HT, hypertension; HDL, high density lipoprotein; LA AP, left atrium anteroposterior; LDL, low density lipoprotein; LVEF, left ventricular ejection fraction; NYHA, New York Heart Association; NT‐proBNP, N‐terminal pro‐brain natriuretic peptide; PCI, percutaneous coronary intervention; sPAP, systolic pulmonary artery pressure; TIA, transient ischemic attack; WBC, white blood cell.

**Figure 1:**
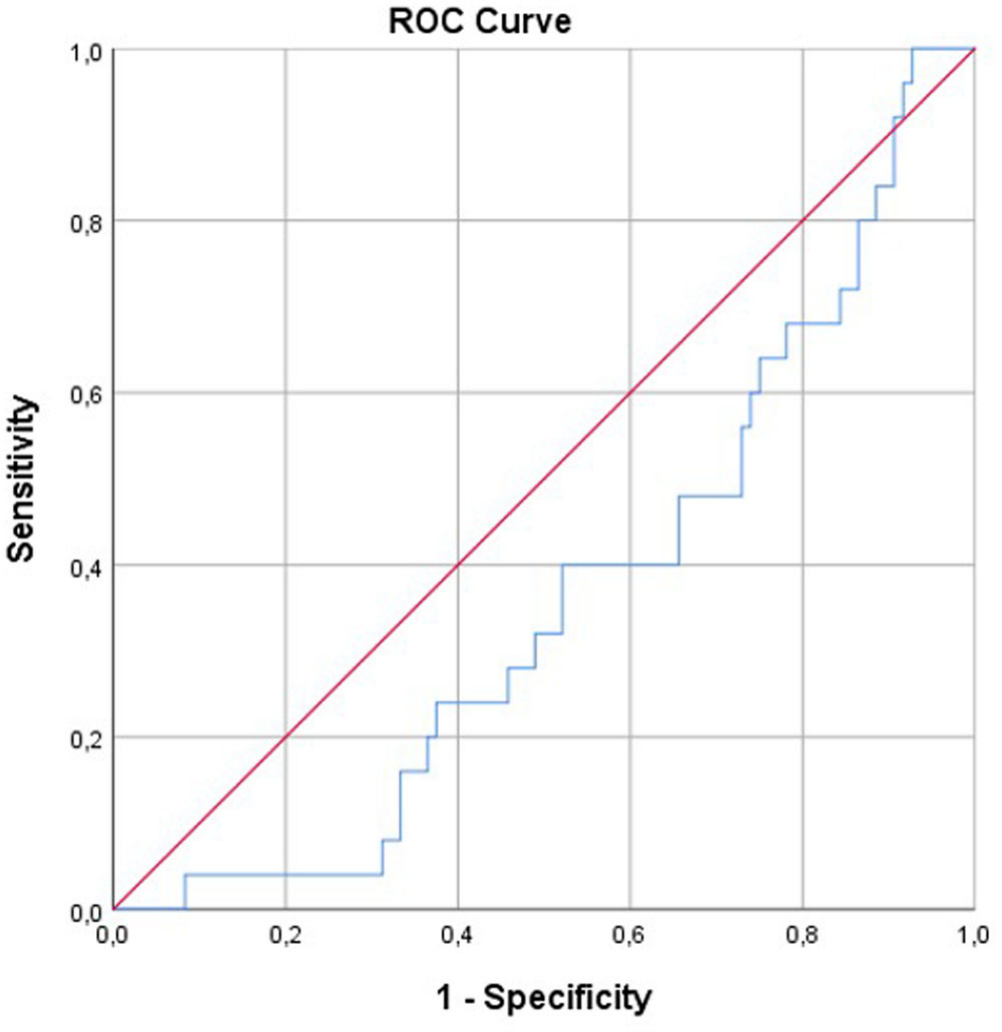
The ROC curve analysis of the HALP score.

A ROC curve analysis was performed to predict the optimal cut‐off value of the HALP score. The are under the curve (AUC), sensitivity, specifity, and the cut‐off value were 0.650, 57%, 43%, 21.5 respectively (*p* = 0.014) (Figure 1).

On univariate analysis, diabetes mellitus, hypertension, serum albumin level, estimated glomerular filtration rate and the HALP score were found to be associated with in‐hospital mortality (Table 2). Variables with a p‐value less than 0.2 with in‐hospital mortality in univariate analysis were incorporated into the multivariate analysis. In multivariate analysis, none of the parameters were found as independently associated with in‐hospital mortality (Table 3).

**Table 2:** The association of potential parameters with in‐hospital mortality in patients with AHF.

	Univariate analysis		
	OR	95% CI	*p* value
Age	1.046	0.998–1.096	0.058
DM	2.452	1.059–5.679	0.036*
HT	2.982	1.052–8.453	0.040*
AF	2.294	0.726–7.252	0.157
WBC count	0.989	0.887–1.103	0.843
Hemoglobin	0.876	0.754–1.018	0.083
Platelet count	1.001	0.997–1.006	0.609
Lymphocyte count	0.774	0.467–1.282	0.320
Albumin	0.352	0.135–0.919	0.033*
HALP score	0.977	0.957–0.996	0.020*
eGFR	0.972	0.953–0.992	0.005*
C‐reactive protein	1.006	0.992–1.019	0.427
NT‐proBNP	1	1‐1	0.805

*Note:* Bold values represent the statistically significant results.

Abbreviations: AF, atrial fibrillation; DM, diabetes mellitus; eGFR, estimated glomerular filtration rate; HT, hypertension; NT‐proBNP, N‐terminal probrain natriuretic peptide; WBC, White blood cells.

**Table 3:** The multivariate analysis for in‐hospital mortality in patients with AHF.

	Multivariate analysis		
	OR	95% CI	*p* value
Age	1.027	0.974–1.083	0.323
DM	2.545	0.949–6.828	0.064
HT	1.731	0.538–5.575	0.357
AF	2.792	0.802–9.715	0.107
HALP score	0.981	0.958–1.004	0.098
eGFR	0.984	0.963–1.007	0.172

## 4 Discussion

The present study aimed to investigate the relationship between a novel index, the HALP score, and in‐hospital mortality in patients with acute heart failure. Our findings demonstrated that lower HALP scores were associated with a higher likelihood of in‐hospital mortality among patients with acute heart failure. Notably, the HALP score exhibited a predictive value for in‐hospital mortality in patients with AHF, with a sensitivity of 57% and a specificity of 43%. Specifically, a cut‐off value of 21.5 may be adopted as a reference point for risk stratification in individuals admitted with heart failure.

Malnutrition and inflammation are frequently encountered and often co‐exist in a variety of chronic disease states including chronic renal failure, rheumatoid arthritis, and heart failure. Recently, the coexistence of these conditions has been referred to as the malnutrition‐inflammation complex and exert a significant impact on patient outcomes. The adverse effect of malnutrition‐inflammation complex on prognosis has been extensively evaluated specifically in hemodialysis patients [18]. On the other hand, although both conditions frequently co‐exist in patients with chronic heart failure, research investigating the malnutrition‐inflammation complex in this population remains limited.

The HALP score was initially developed by Chen et al. to predict prognosis in individuals with gastric carcinoma [19]. It involves the four essential markers of immune and nutritional status: albumin, hemoglobin, lymphocytes, and platelets. Hence, the HALP score may serve as a biomarker for both protein‐energy malnutrition and inflammation. While it is primarily utilized to ascertain survival in diverse cancer types, the correlation between the score and a range of cardiovascular diseases has also been proposed. Recently, Karakayali et al. have indicated that the HALP score may serve as an independent predictor of in‐hospital mortality in patients undergoing primary percutaneous intervention for ST‐segment elevation myocardial infarction [20]. The retrospective cohort studies of NHANES database have suggested that higher HALP scores were associated with lower all‐cause and cardiovascular mortality, and the HALP score may be predictive of the prognosis of coronary artery disease [21, 22].

Systemic inflammation is central to the development, progression, and prognosis of heart failure. Likewise, nutritional status is closely linked to heart failure progression, with poor nutritional status frequently observed in the advanced stages of the disease. Therefore, the HALP score—a marker recognized for reflecting both inflammation and nutritional status—may serve as a valuable tool in assessing the prognosis of heart failure. To the best of our knowledge, so far, only two studies have evaluated the role of the HALP score in relation to heart failure. Kocaoglu et al. have examined the prognostic value of the HALP score and the modified HALP score, termed m‐HALP (thrombocytesxalbuminxlymphocytesxhemoglobin), in 101 pateints who were admitted to the emergency department due to exacerbation of heart failure. Their findings indicated that, while the classical HALP score was not associated with either 1‐week or 3‐month mortality, the m‐HALP score was significantly higher in surviving patients [16]. In the abovementioned study, blood platelet levels were notably lower and within the thrombocytopenic levels in patients who died during follow‐up. In our study, however, there was no meaningful difference in blood platelet levels between survivors and non‐survivors. We believe that this discrepancy in blood platelet levels may explain the differences in findings between the two studies. In a very recent retrospective cohort study by Liu et al., the HALP score was found to be predictive of both short‐ and long‐term mortality in patients hospitalized for heart failure [23]. Likewise, we identified a correlation between lower HALP scores and increased in‐hospital mortality rates among patients requiring hospitalization due to acute heart failure.

## 5 Limitations

Our study is subject to several limitations. To begin with, it is a single‐centre study involving a relatively low number of patients. Second, we only analyzed in‐hospital mortality and did not conduct long‐term follow‐up of the patients. In addition, the retrospective design of the study reduces its statistical power. While the present study is valuable in demonstrating, for the first time the relationship between the HALP score and in‐hospital mortality in heart failure, the utility of the score in daily clinical practice may be limited due to the rather low AUC value. In the future, larger‐scale follow‐up studies are needed to validate the predictive value of the HALP score with regard to mortality among individuals with heart failure.

## 6 Conclusion

Despite all therapeutic efforts, in‐hospital mortality rates for patients with acute heart failure remain high. Consequently, it is of paramount importance to timely recognize the patients who would have an unfavorable prognosis during admission, to timely and intensively implement the therapeutic interventions. The HALP score, being a readily calculable tool, serves as an effective means to pinpoint individuals at a heightened risk of in‐mortality. We believe that the HALP score holds promise as a practical tool for predicting mortality among patients admitted for HF.

## Author Contributions

**Akbulut Mugea:** analysis and interpretation, drafting of the paper, writer. **Izci Cenana:** conception, design, data collection. **Ozyuncu Nila:** data collection, literature review, critical review, revising the manuscript critically for intellectual content. **Esenboga Ka:** critical review, revising the manuscript critically for intellectual content. All authors have made a sufficient contribution to the work.

## Conflicts of Interest

The authors declare no conflicts of interest.

## Data Availability

Raw data that support the findings of this study are available from the corresponding author, upon reasonable request.
